# scToppR: a coding-friendly R interface to ToppGene

**DOI:** 10.1093/bioinformatics/btae582

**Published:** 2024-09-28

**Authors:** Bryan Granger, Stefano Berto

**Affiliations:** Bioinformatics Core, Department of Neuroscience, Medical University of South Carolina, Charleston, SC 29425, United States; Neurogenomics Laboratory, Department of Neuroscience, Medical University of South Carolina, Charleston, SC 29425, United States

## Abstract

**Motivation:**

The scToppR package provides a ToppGene interface from R programs/scripts to fully access/control the database for functional enrichment without the need for active interaction on its Web site (https://toppgene.cchmc.org/).

**Results:**

The library facilitates the functional enrichment analysis and visualization by interacting with ToppGene, downloading the functional enrichment dataframes, and using R environment to visualize the final results.

**Availability and implementation:**

Code and documentation are currently available at https://github.com/BioinformaticsMUSC/scToppR

## 1 Introduction

Gene set enrichment analysis has become a fundamental component of bioinformatic pipelines, enabling researchers to interpret high-throughput experiments. Genes representing a common biological processes are stored and maintained in databases such as Gene Ontology, the Reactome pathway database, the Kyoto Encyclopedia for Genes and Genomes (KEGG), and the Molecular Signatures Database (MSigDB, Ashburner *et al.* 2000, Kanehisa *et al.* 2010, Liberzon *et al.* 2015, Milacic *et al.* 2024). These databases host thousands of gene set collections, curated from existing scientific literature or derived from molecular experiments. Pathways databases are accessible by Application Programming Interface (API), which facilitate access and querying. Among web platforms dedicated to gene set enrichment, ToppGene Suite (http://toppgene.cchmc.org) is a free and open tool focused on gene list functional enrichment and candidate gene prioritization (Chen *et al.* 2009). This tool is widely used to quickly access functional categories and candidate gene prioritization with more than 2000 citations. Although the web site is user-friendly and convenient to use, researchers cannot seamlessly integrate it into a code-based workflow. To address this challenge and incorporate visualization, we developed a package to interface R and the ToppGene web service API.

We developed a versatile R library called scToppR to access ToppGene’s functional enrichment capabilities. scToppR is an R wrapper that provides easy access to ToppGene’s publicly available API endpoints, simplifying pathway enrichment analysis and visualization for single cell RNA-seq and bulk RNA-seq differential expression results. The package bridges the simplicity and thoroughness of ToppGene with existing R-based workflows.

The primary function for scToppR, “toppFun(),” takes as input a dataframe from differential gene expression analysis, such as the outputs of functions like Seurat’s “FindMarkers()” or Libra’s “run_de()” (Hao *et al.* 2021, Squair *et al.* 2021). The dataframe only requires columns indicating the specific gene being tested, the group, such as cluster or cell type or class, in which the gene is being tested, the average log fold change of the gene, and the *P*-value or adjusted *P*-value denoting the significance of the change in expression. The “toppFun()” function allows users to specify the names of these columns without altering the dataframe. Alternatively, the raw dataframe can be provided, and users can set thresholds (if any) for significance, fold change, and the number of genes to include in the enrichment test. The package executes this query via a representational state transfer (REST) request using the R package “*httr*” (Khalil and Fakir 2017).

With this input data and specific parameters, “toppFun()” creates a query for toppGene, sending the relevant lists of genes for each group and compiling all results into one single dataframe. Users can perform enrichment analysis for all ToppGene categories or a specified subset and can also specify parameters for ToppGene, such as the minimum genes in a term or the number of results per group per category. The resulting dataframe includes all data from these enrichment tests, such as gene set names, sources, URLs (if applicable) and statistics like number of genes in each term and adjusted *P*-values. This dataframe can be saved as one single large table or automatically saved into separate files for each group of cells.

With the results, users can quickly create dotplots and balloon plots to visualize the functional enrichment. The “toppPlot()” function generates one or multiple dotplots for specific clusters within each category. The plots display the top results for each combination of group and category, with the ratio of genes from the query and the resulting gene sets on the x-axis and the top pathways on the y-axis. The size of the dot represents the number of genes from the query found in each functional category, and the color depicts the negative log10 of the adjusted *P*-value of the enrichment test. The visualizations are created with “ggplot2,” allowing easy customization once generated (Wilkinson 2011).

The “toppPlot()” function allowed users to plot the results for multiple groups or clusters of cells simultaneously. By including multiple groups in the function call, it returns a list containing all the desired plots. Alternatively, users can opt for a list of ggplot elements. Additionally, the save parameter in the “toppPlot()” function can be used to automatically generate PDF versions of each individual plot.

scToppR also includes a “toppBalloon()” function that creates a balloon chart plot for one category encompassing all available clusters or groups of cells. This visualization displays the top three terms for each cluster, with the shape and color of the dots representing the gene ratio and the significance, respectively. This approach effectively highlights similarities or differences between different cell types or clusters in a single plot.

Overall, the package enables users to quickly assemble and aggregate data from ToppGene entirely within a code-based workflow. While the ToppGene website is user-friendly and easy to navigate, scToppR allows researchers to effortlessly submit gene lists directly from differential expression analyses to ToppGene, including in an automated manner, thus minimizing the time required for analysis. With the aggregated results from scToppR, users can also efficiently plot and save results from any combination of ToppGene categories and clusters or cell types in their data. In short, scToppR offers a quick and straightforward way to collect and analyze ToppGene in R.

## 2 Example application

For input, the package requires a dataframe of differentially expressed genes, including clusters or other specific classifications, log fold changes, and adjusted *P*-values. Since the user can specify the column names in the “toppFun()” function call, it is not necessary to have these exact column names in the dataframe. This makes the package more flexible, allowing it to accept input without requiring specific preprocessing of the data. To show the capabilities of scToppR, we have included two vignettes: a simple introduction of the workflow and one that follows differential gene expression analysis. For the differential expression example, we have provided a sample dataset within the package, which is derived from interferon-β (IFNB) treated and control peripheral blood mononuclear cells (PBMC) (Kang *et al.* 2018) and is also available as part of the R package “SeuratData.” The dataset in scToppR includes results from a Wilcoxon test comparing control and treated cells, using the Seurat “FindMarkers” function (Hao *et al.* 2021). To access the dataset in scToppR, use the function “data(‘ifnb.de’)” (Table 1).

**Table 1.** Example dataframe input for scToppR.

	p_val	avg_log2FC	pct.1	pct.2	p_val_adj	celltype	gene
1	1.61E−04	6.898413	0.047	0	1.00E+00	NK	GMPR
2	1.90E−07	6.893728	0.052	0	2.68E−03	CD16 Mono	HRASLS2
3	2.74E−25	6.888089	0.468	0.005	3.85E−21	B Activated	IFIT2
4	2.11E−06	6.887607	0.084	0	2.96E−02	DC	HRASLS2
5	0.00E + 00	6.883785	0.992	0.056	0.00E+00	CD14 Mono	IFIT3
6	4.50E−04	6.882053	0.03	0	1.00E+00	B	MS4A12

With this dataset, users can easily submit a “toppFun()” query by specifying “cluster_col = “celltype”, gene_col = “gene”, p_val_col = “p_val_adj”,” and logFC_col = “avg_log2FC.” Columns with different names will not be used. Additionally, “toppFun()” can filter and sort records based on log fold change or adjusted *P*-values. Thus, a researcher’s differential gene expression data does not need to be preprocessed, sorted, or filtered before running “toppFun().”

To run a “toppFun()” query with the above dataframe (named ifnb.de, which is included in the package), use the following command line:

*toppData <- toppFunn(ifnb.de, cluster_col = “cluster”, gene_col = “gene”, p_val_col = “p_val_adj”, logFC_col = “avg_log2FC”)*

To visualize the results, a researcher can simply use the following code:

*toppPlot(toppData, category = “GeneOntologyMolecularFunction”, clusters = 0)*

**Figure 1.**
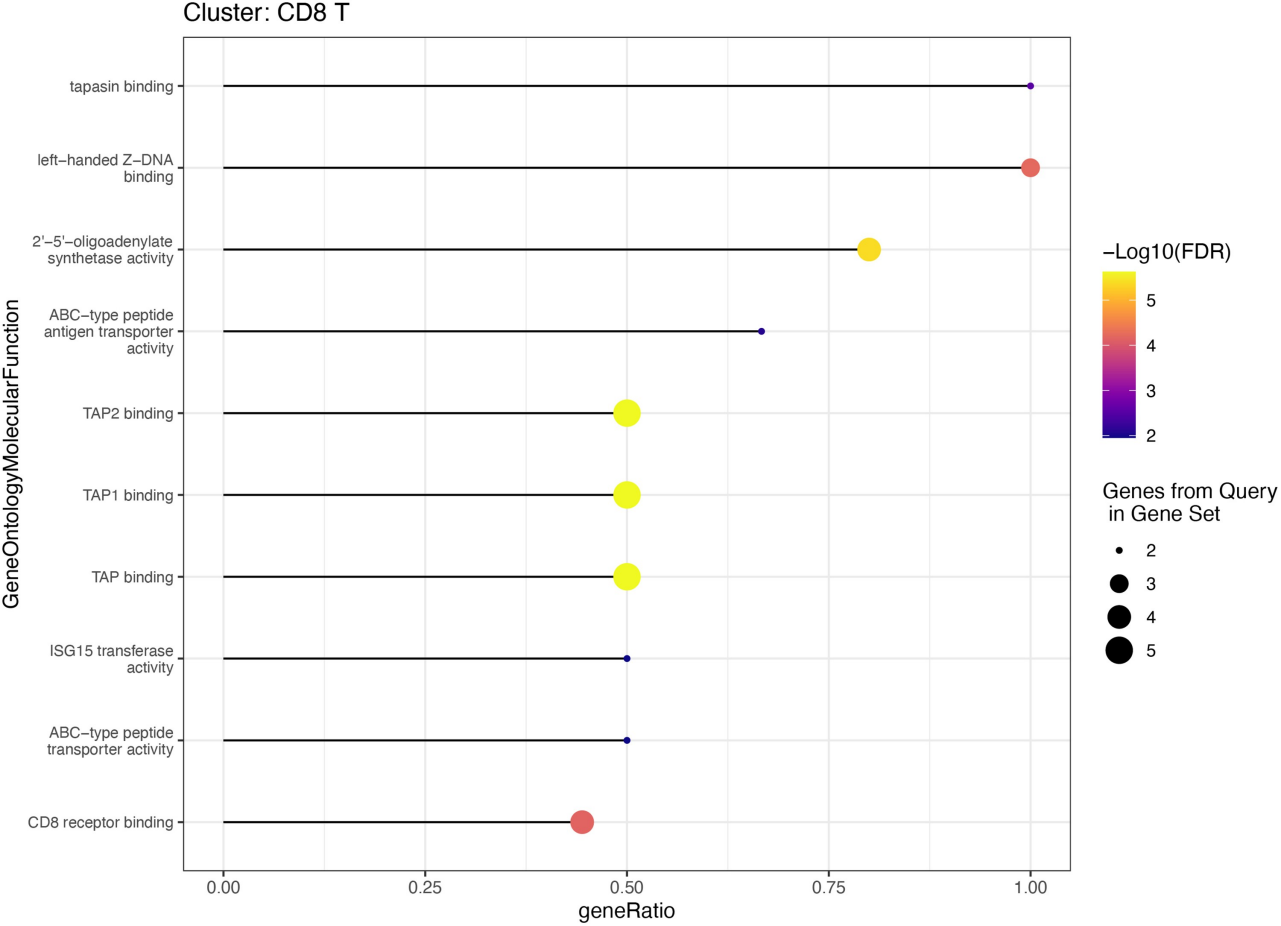
toppPlot. A dotplot showing the results from ToppGene in the category GeneOntologyMolecularFunction for a subset of cells from the example dataset.

In this example, the code generates a dotplot for the celltype “CDT 8” using the results from the ToppGene category “GeneOntologyMolecularFunction” (Fig. 1).

A balloon plot showing the top gene ontology terms from each cell type for a given ToppGene category can also be created by executing the following code:

*toppBalloon(toppData, categories = “GeneOntologyBiologicalProcess”, balloons = 2)*

**Figure 2.**
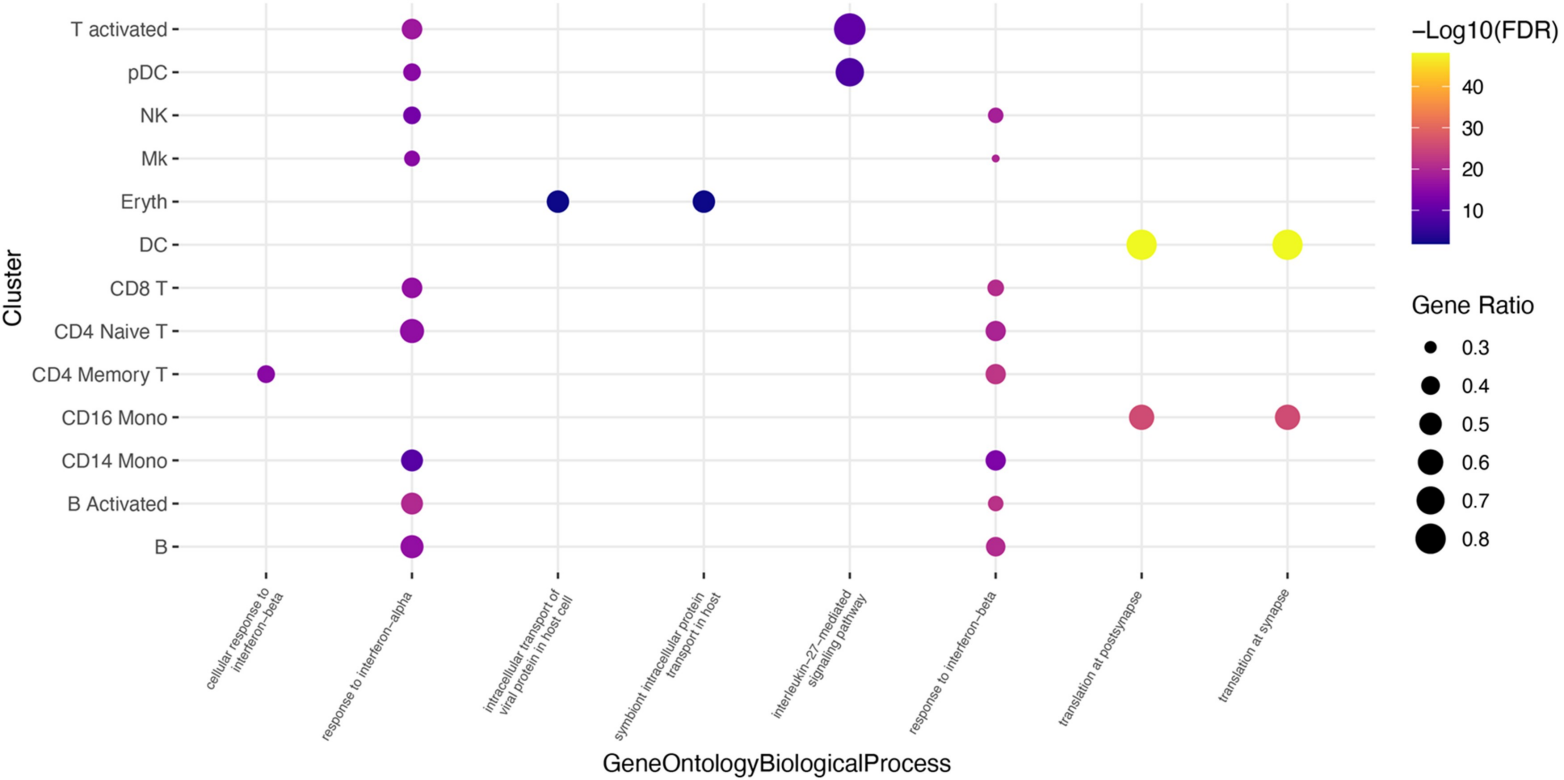
toppBalloon plot. A balloon plot showing the top terms for each celltype from the ToppGene results in the GeneOntologyBiologicalProcess category.

The code generates a balloon plot for the ToppGene functional category “GeneOntologyBiologicalProcess.” The number of dots per cluster can be modified with the balloons parameter (Fig. 2).

The plotting functions can be used with any category available in ToppGene. Functional categories can be omitted if the results do not meet specific thresholds, or users can modify them using the function parameters. To easily fetch the list of ToppGene functional categories in R, simply run “get_ToppCats().”

The scToppR package also includes convenient file-saving functions. With “toppSave(),” users can easily save the toppData as an Excel spreadsheet, CSV, or TSV file. The function can also automatically split the data into tables for each cell type, saving them individually.

## 3 Conclusion

The package scToppR aims to connect researchers ToppGene’s functionalities via a code-based workflow and to simplify the resulting visualizations. We plan to develop and update scToppR as needed to support quick gene ontology analyses. scToppR is currently available as an open-source package via Github (https://github.com/BioinformaticsMUSC/scToppR), and Bioconductor. The package is released under the MIT license, subjected to the terms of use of ToppGene (https://toppgene.cchmc.org/navigation/termsofuse.jsp).

## Data Availability

The IFNB dataset can be accessed via the Gene Expression Omnibus with accession number GSE96583 and can be downloaded via the R package SeuratData (https://github.com/satijalab/seurat-data). The PBMC data also can be accessed via the SeuratData package.
